# Complications of Stem Cell–Based Injections for Knee Osteoarthritis: A Systematic Review

**DOI:** 10.1177/15563316241271058

**Published:** 2024-08-16

**Authors:** Clara Riggle, Maddison McLellan, Hunter Bohlen, Dean Wang

**Affiliations:** 1 Department of Orthopaedic Surgery, University of California, Irvine, Orange, CA, USA

**Keywords:** knee osteoarthritis, stem cell injections, safety

## Abstract

Knee osteoarthritis (OA) remains a common cause of knee pain and dysfunction. Stem cell–based injections have been widely used for the treatment of knee OA, but the types and rates of post-injection complications are not well characterized. We sought to characterize the type and severity of adverse events and quantify the frequency of adverse events associated with stem cell injections used to treat knee OA. We conducted a systematic review that followed the Preferred Reporting Items for Systematic reviews and Meta-Analyses (PRISMA) guidelines. We searched the PubMed and the Cochrane library databases for studies on adverse events and complications associated with stem cell–based therapies used to treat knee OA published from January 2000 through June 2021. Inclusion criteria were the use of intra-articular autologous bone marrow stem cells (BMSCs) or bone marrow aspirate concentrate (BMAC), autologous adipose-derived mesenchymal stem cells (ADMSCs) including microfragmented lipoaspirate, concentrated adipose tissue, cultured stem cells, autologous stromal vascular fraction (SVF), or umbilical or placental derived stem cells in human participants. Primary data extracted from included studies were patient demographics, methods of treatment, and reported character, duration, and severity of adverse events. A total of 427 studies were screened, and 48 studies were included, including randomized controlled trials, prospective studies, and retrospective studies. Among the 1924 patients in the analysis, there was an overall 12.3% rate of transient adverse events, the most frequent being swelling and pain at the injection site. Umbilical cord–derived (51.7%) and cultured ADMSC (29.5%) injections had a significantly higher occurrence of these adverse events than BMSC and SVF injections. No other adverse events, including infection, fat embolism, or medical complications, were reported. Despite significant heterogeneity of the included studies in terms of the protocol, formulation, timing, and location of injections, the findings of this systematic review suggest that, in the short term, treatment of knee OA with autologous mesenchymal stem cell injections poses no risk of major complications (infection, sepsis, neoplasm, embolism, or death) and poses moderate risk of swelling and pain at the injection site lasting less than 4 weeks. Further long-term studies are needed to conclusively determine the safety profile of these injections.

Osteoarthritis (OA) is the most common cause of degenerative joint disease in the United States, with the knee being the most common joint affected by loss of function, pain, and stiffness [21]. Current nonoperative treatments include exercise, physical therapy, oral anti-inflammatory medications, and intra-articular injections of corticosteroids, platelet-rich plasma (PRP), or hyaluronic acid (HA). The gold standard treatment for knee OA consists of exercise modification, physical therapy, and corticosteroid injections—with or without HA injections. Knee arthroplasty is the gold standard for patients who have failed conservative treatment, which can only slow cartilage degeneration. However, arthroplasty presents many postoperative complications, posing the need for novel treatment methods [52]. Thus, research into the use of stem cell–based therapies, both alone and in conjunction with current knee OA treatment methods, has grown in the last decade [38].

To date, some studies on stem cell–based treatments for treating knee OA have reported reduced pain, improved function, and delayed operative intervention [4,8,43]. Treatments include injections of progenitor cells derived from stromal vascular fraction (SVF), adipose, bone marrow, or umbilical cord blood [1,4,12,13,15,18]. Some of these treatments involve techniques that expand cells or alter the microcomposition of the cells prior to injection, including cell culture, microfractionation, and collagenase digestion [20,26,58]. There have also been outcomes reported on the use of stem cell therapy in combination with other therapies, including knee arthroscopy and associated procedures, as well as HA or PRP injections [1,12,18,28,48].

With the wide range of sources, preparations, and applications for stem cell–based treatments for knee OA, the risks of these treatments are unclear [12,13,15]. As a result, a consensus on the general safety of stem cell therapies for knee OA has not been established, and the various stem cell–based treatments are not regulated by the US Food and Drug Administration (FDA). As the population of patients with knee OA continues to grow, the clinical utilization of stem cell–based treatments for knee OA is expected to increase. Therefore, it is important to characterize the risks and safety profiles of these treatments [38].

The goal of this systematic review was to characterize and quantify the reported adverse events and complications associated with stem cell–based therapies used to treat knee OA. The secondary goal was to identify differences in character, frequency, or severity of adverse events associated with stem cell injections derived from different sources. Our specific research questions were: What is the frequency of adverse events associated with intra-articular stem cell injections for knee OA? What is the character of these adverse events, as described by severity, type of symptom, and time course? Does the frequency or character of the adverse events differ based on the source of the stem cell injection? Do these injections show increased rates of adverse events when paired with surgery, HA, or PRP injections?

## Methods

### Search Strategy

This systematic review followed the Preferred Reporting Items for Systematic reviews and Meta-Analyses (PRISMA) guidelines (http://www.prisma-statement.org/). A thorough search of the existing literature was performed for all complications and adverse events related to stem cell–based treatments for knee injuries and pathologies. The PubMed and Cochrane Library digital databases were searched using the keywords “AD-MSC,” “BM-MSC,” “adipose derived stem cells,” “stromal vascular fraction,” “multipotent mesenchymal stem cells,” and “knee.” In addition, the references from any reviews and meta-analyses found in this method were reviewed to identify any additional studies fitting the inclusion criteria.

### Eligibility Criteria

Our inclusion criteria were clinical studies published from January 2000 through June 2021 reporting on the use of intra-articular autologous bone marrow stem cells (BMSCs) or bone marrow aspirate concentrate (BMAC); autologous adipose-derived stem cells (ADMSCs) including microfragmented lipoaspirate; concentrated adipose tissue; cultured stem cells; autologous SVF; and umbilical or placental derived stem cells in human participants. Only stem cell–based intra-articular injection treatment was included, and any treatment involving implanted scaffolds were excluded. We excluded studies with fewer than 10 participants and those published in a language other than English. In addition, animal studies, reviews, and responses or letters to the editor were excluded, as were studies that included pathology outside of knee OA.

### Data Extraction

Study abstracts were screened using the inclusion and exclusion criteria, and for those with abstracts fitting our inclusion criteria, full text articles were screened. Analyzed studies were separated into 3 categories: stem cell–based injections alone, stem cell–based injections with PRP and/or HA, and stem cell–based injections with surgical intervention. Studies that used multiple sources of stem cell–based biologics were analyzed separately, as were studies pairing surgery and biologics. The reports were split into these categories and qualitatively analyzed separately. This was done to account for the effects from adjunctive surgical or orthobiologic procedures, as well as any effects from the combinations of these treatments.

### Outcomes of Interest

Our primary outcomes of interest were the specific type, number, and symptom duration for all reported adverse events. Additional outcome measures included patient demographics and methods of treatment. When evaluating methods of treatment, stem cell harvest location, injection location, concentration/preparation of cells, and patient population were noted. This review addressed any adverse events reported in studies, to the degree of detail specified regarding duration and characteristic. Adverse events were defined as any unintentional and unfavorable medical effect reported within the study, including non-severe short-term adverse events such as stiffness, weakness, swelling, and pain lasting less than 4 weeks; non-severe long-term adverse events such as pain and swelling lasting longer than 4 weeks; and severe adverse events such as infection, sepsis, neoplasm, embolism, and death. All statistical analysis was performed in the R platform, with significance level *P* = .05.

### Risk of Bias Assessment

Studies were evaluated for quality using the Methodological Index for Non-Randomized Studies (MINORS) criteria and the revised Cochrane risk-of-bias tool (RoB 2) for randomized trials [53]. The perfect MINORS criteria score for non-comparative studies is 16 and for comparative studies is 26. The RoB 2 rates bias as low risk, high risk, or some concern. Two authors evaluated each study using the appropriate assessment tool.

## Results

**Fig. 1.**
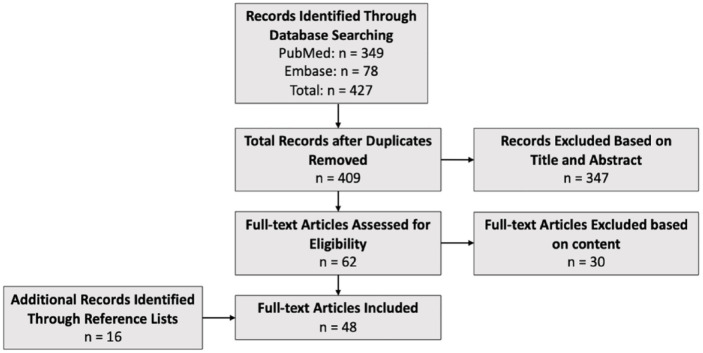
PRISMA flow diagram of included studies.

The initial literature search identified 445 studies. After screening, 48 studies involving 1924 patients matched our criteria. Of the 48 studies included, 30 used stem cell–based therapies alone, 12 used stem cell–based therapy in adjunct to surgery, and 6 used stem cell–based therapy in adjunct to PRP or HA injections (Fig. 1). Of note, many studies did not include a specific time frame in which adverse events occurred, nor did they note duration. Similarly, many studies did not report details of adverse events or complications. All studies used in this review were level of evidence II through IV.

Studies were further analyzed based on formulation type: 14 studies used cultured ADMSC (including microfragmented lipoaspirate and cultured stem cells), 15 used SVF, 15 used BMSC, 2 used allogeneic stem cells derived from umbilical cord blood (UC-SC), 1 used both bone marrow and ADMSC, and 1 used SVF and ADMSC (Supplemental Table 1). A majority of studies were randomized controlled trials (RCTs), case-control studies, large case series, or cohort studies.

Of the 48 studies in our review, 30 investigated the results of isolated stem cell–based injection for knee OA. These studies included 1152 patients aged 18 to 80 years. Sample sizes ranged from 10 to 21, with an average sample size of 33.3. Seven of the studies used ADMSC [11,16,17,30,31,42,51,59], 9 used SVF [3,13,19,20,29,37,46,57,61], 10 used bone marrow–derived cells [1,2,6,12,15,40,45,48,50,54], and 2 used allogeneic umbilical cord–derived cells [24,34]. One study compared SVF and ADMSC and 1 study compared BMAC and ADMSC [35,61]. Final follow-up ranged from 6 months to 3 years after initial injection. Fourteen of the studies reported stem cell counts that were within injection protocols, ranging from 2 × 10^6^ to 1 × 10^8^ [1,6,11–13,16,17,29–31,34,37,40,50,51,57,61]. Injection protocols ranged from single injections to a series of 2 to 3 injections, taking place over intervals between 3 and 48 weeks apart [51].

Among the 1152 patients receiving isolated stem cell injections, the rate of reported adverse events was 14.9%. The rates of adverse events in the various stem cell source groups were 29.5%, 10.7%, 51.7%, and 8.1% for cultured adipose, bone marrow, umbilical cord, and SVF, respectively (Table 1). Compared with other sources, umbilical cord (51.7%) and cultured ADMSC (29.5%) injections had the highest rate of adverse events (*P* < .01).

**Table 1.** Total adverse events by stem cell source.

Parallel intervention	Stem cell source	Number of studies	Number of patients	Number of AE	Number of SAE	Rate of AE	Rate of SAE
None	Adipose (cultured)	9	244	72	0	29.50%	0%
None	Bone marrow	11	533	57	0	10.70%	0%
None	Umbilical cord	2	29	15	0	51.70%	0%
None	SVF	10	346	28	0	8.10%	0%
HA and/or PRP	Bone marrow	3	68	15	0	22.10%	0%
HA and/or PRP	SVF	3	345	37	0	10.70%	0%
Surgery	Adipose (cultured)	6	191	6	0	3.10%	0%
Surgery	Bone marrow	2	74	0	0	0.00%	0%
Surgery	SVF	3	94	6	0	6.40%	0%
Combined total		48	1924	236	0	12.26%	0%

Two studies with no parallel intervention used stem cells from different sources, in different patients. These patients and AE were split up, respectively.

The vast majority of the adverse events lasted less than 4 weeks, with the most common adverse event being swelling and pain at the site of injection. Other transient adverse events included pain and swelling at harvest site for the adipose, SVF, and bone marrow–derived cells groups. In the ADMSC group, 2 reported adverse events—pain and/or swelling at the injection site [10]—lasted longer than 4 weeks. In the BMAC group, 1 study showed 4 cases of swelling that lasted longer than 4 weeks [48]. In the SVF and umbilical cord–derived groups, no adverse events lasting longer than 4 weeks were reported.

Six studies paired stem cell–based injections with concurrent PRP or HA injections. Sample size from studies in the PRP/HA subgroup ranged from 12 to 242 and included a total of 413 patients, ages 18 to 80 years. Five of these studies used PRP [4,28,36,41,49], and 1 study used HA for concurrent injection [28]. One of the PRP studies described concomitant injection of “platelet rich in growth factors (PRGF)” [42]. The follow-up time was at least 6 months for all studies. Four of the studies reported cell counts, with a range from 1.7 × 10^6^ to 1 × 10^8^ (Supplemental Table 1) [4,27,28,49].

Among the 413 patients in this group, a 12.6% rate of adverse events was reported, with 52 cases noted. No adverse events lasting longer than 4 weeks were reported. No complications that led to further surgical intervention or hospitalization were noted. The rate of adverse event was 22.1% in the bone marrow–derived group and 10.7% in the SVF group. The most common adverse event was articular pain at the injection site (n = 25) and included articular pain within 24 hours of infiltration [4,27,41].

Twelve studies investigated stem cell–based injections combined with surgical procedures and included 359 patients aged 18 to 75 years. Concurrent surgical treatments included diagnostic arthroscopy, arthroscopic debridement, arthroscopic microfracture, and high tibial osteotomy [25,33,44,56,60]. Six studies used adipose-derived stem cells (ADSCs) [5,11,25,33,44,47], 2 studies used BMSC [55,60], 3 studies used SVF [18,23,56], and 1 study used both BMSC and ADMSC together [47]. Six studies performed cell counts of the stem cell injections, with a range of 1 × 10^6^ to 1 × 10^8^ [11,18,23,25,44,56]. Minimum follow-up time ranged from 6 to 36 months, and sample sizes ranged from 10 to 52 patients [44,47]. All surgical procedures were performed for an indication of knee OA, and all injections were performed intraoperatively.

Among the 359 patients in this group, there was a 4.7% rate of adverse events reported, with 12 total cases noted. No adverse events were reported lasting longer than 4 weeks. The rates of transient adverse events in each stem cell–based source group were 3.1%, 0%, and 6.4%, for cultured adipose, bone marrow, and SVF, respectively. All adverse events lasted less than 4 weeks, the most common being injection site pain and swelling.

Regarding bias assessment, non-randomized studies evaluated using the MINORS criteria all had scores >10, a majority between 12 and 15, indicating moderate to good quality (Supplemental Table 2). In the RCTs evaluated using the RoB 2, 2 studies showed high risk of bias, 3 studies showed some concern, while the remaining showed low concern for bias (Supplemental Table 3). Risk-of-bias assessment was performed on the 48 studies used in the analysis, with evidence level II to IV. Areas of possible bias include attrition bias and performance bias.

## Discussion

This systematic review found an absence of major complications reported in the short term with stem cell–based injections for knee OA. However, within all treatment groups, minor transient adverse events were noted, regardless of harvested stem cell source or concurrent therapy. An overall combined adverse event rate of 12.3% was observed among treatment groups; the most frequently reported was short-term pain or swelling at the injection site. Most transient adverse events resolved within 1 week, suggesting that adverse events may not be indicative of long-term tissue damage or morbidity, but long-term studies are needed [17,19,30,31,51]. In addition, no serious complications such as joint infection, sepsis, neoplasm, embolism, or death were noted in any study included in this review, but again further study is needed into the long-term complications of stem cell–based injections for knee OA.

This study has several limitations. First, treatment protocols in the included studies displayed significant heterogeneity, including variations in injection protocol, formulation, timing, and location. Second, reporting of timing, character, and rate of adverse events varied significantly among studies, and thus statistical conclusions could not be drawn on the rates of these issues among treatment groups. This presents an opportunity for future research to compare rates and characteristics of adverse events depending on the formulation of stem cell–based injection treatment. Third, given the novelty of treatment, minimal long-term data was available to evaluate for potential late complications, such as neoplasm. Finally, the studies used in this review present some risk of bias, especially attrition and performance bias. As a review of heterogeneous studies including RCTs, cohort studies, case-control studies, and large case series, a significant proportion of the studies are level III or level IV evidence. Further long-term studies will be needed to conclusively evaluate the safety profile of these injections.

Among the stand-alone injections groups, observed rates of transient adverse reactions for adipose-derived, bone marrow–derived, SVF-derived, and umbilical cord–derived stem cell groups were 29.5%, 10.7%, 8.1%, and 51.7%, respectively. The data suggest that ADMSC and umbilical cord–derived stem cells may be associated with higher rates of short-term reactions. Of note, only the stand-alone stem cell injection group reported pain and swelling lasting longer than 4 weeks, with rates of 0.82% and 0.75% in the adipose-derived and bone marrow–derived groups, respectively [10,48].

For the treatment group in which stem cell–based injections were given in conjunction with PRP or HA injections, rates of adverse events were higher than those reported for isolated stem cell–based therapy, suggesting that multiple injections may increase the rate of local soft-tissue swelling and pain. In isolation, both PRP and HA injections have been shown to cause inflammation, pain, and effusion in the knee [39]. However, no adverse events were reported to last longer than 4 weeks, and no serious complications were reported.

No substantial increase in the rate of adverse events was reported when the stem cell–based injections were paired with surgical procedures, with rates ranging from 0% for bone marrow to 6.4% in studies using SVF. Of note, most adverse events reported in the injection groups were for pain and swelling at the injection site, which would be difficult to isolate from symptoms associated with concurrent surgery. No long-term adverse reactions or increased rate of surgical complications were reported when concurrent stem cell injection was performed, suggesting that stem cell–based injections may be administered safely in conjunction with surgery; however, more research and higher level data are needed on this topic.

Across all modes of treatment and stem cell formulations, no reports of serious complications such as sepsis, infection, neoplasm, hospitalization, or death were described for nearly 2000 patients. This reflects the largest review on the topic to date and demonstrates that these injections may be safe to use in the treatment of knee OA. Unfortunately, given the poor characterization of short-term adverse events in the literature, no statistical conclusions could be drawn regarding the relative rates of these events between groups. Dedicated research comparing short-term reactions to various injections is needed to definitively characterize the relative profile of these reactions. Previous studies have found that the rate of serious complications following biologic injections, such as joint infections, is higher than anticipated, while others have found no serious complications following patients up to 2 years [7].

Underreporting and minimization of risks in the current literature can lead to dangerous consequences for patients treated with stem cell–based therapies [9]. Honest reporting of complications and adverse events resulting from stem cell–based injections is crucial to ascertain their effectiveness and risk profiles; clinical utilization currently outpaces the scientific evidence. Eliasberg et al [7] reported on 14 patients across the United States who experienced infection or sterile inflammatory responses resulting from intra-articular injections, with umbilical cord blood being the most common source of these infections. That study discussed a lack of standardization and oversight as potential contributing factors to the serious complications observed in off-the-shelf stem cell–based treatments. Other studies have shown that stem cell–based injections demonstrate low risk, similar to the findings of this study. Song et al reported on the efficacy and safety of stem cell–based injections for knee OA. The review evaluated RCTs, cohort studies, and retrospective studies, including 584 patients and 15 studies [52]. No significant complications were reported across all studies. Like this present study, Song’s review noted reports of transient adverse events such as pain, bleeding, swelling, and additional symptoms at the injection site. Meta-analysis found no statistical difference in the frequency of these adverse events between control and treatment groups. Gobbi et al [14] reported on adverse events of autologous microfragmented adipose tissue injections with long-term follow-up, finding a rate of 12.5% short-term bruising lasting less than 3 days, and no major adverse event on 2-year follow-up. Jiang et al published a meta-analysis evaluating 9 randomized controlled trials using mesenchymal stem cell (MSC) injections from multiple sources to treat knee OA. This study demonstrated a similar safety profile between MSCs harvested from different sources, and no statistical difference in minor adverse events between control groups and treatment groups. One case of infection was reported in this study, which was characterized as a significant adverse event [22]. When comparing the safety of stem cell–based injections with other commonly used injection, stem cell–based injections appear to pose no increased risk. Meta-analysis review of adverse event rates with other commonly used injections by Ma et al [32] found adverse event rates for HA injections of around 27.7%, and event rates for corticosteroid injections of around 14.3%.

In conclusion, this systematic review of 48 studies found nearly 2000 patients treated with stem cell–based injections, with no reports of serious complications such as sepsis, infection, neoplasm, hospitalization, or death. This result was observed when stem cell–based injections were given in isolation and when combined with concurrent surgery or HA/PRP injections. The rate of transient adverse events across all studies was 12.3%, and the highest rate of adverse events occurred after isolated umbilical cord (51.7%) and cultured ADMSC (29.5%) injections. This review suggests that the treatment of knee OA with autologous mesenchymal stem cell injections is safe in the short term; however, further studies are needed to better quantify their short-term adverse events and confirm long-term safety.

## Supplemental Material

sj-docx-1-hss-10.1177_15563316241271058 – Supplemental material for Complications of Stem Cell–Based Injections for Knee Osteoarthritis: A Systematic Review Supplemental material, sj-docx-1-hss-10.1177_15563316241271058 for Complications of Stem Cell–Based Injections for Knee Osteoarthritis: A Systematic Review by Clara Riggle, Maddison McLellan, Hunter Bohlen and Dean Wang in HSS Journal®

sj-docx-2-hss-10.1177_15563316241271058 – Supplemental material for Complications of Stem Cell–Based Injections for Knee Osteoarthritis: A Systematic Review Supplemental material, sj-docx-2-hss-10.1177_15563316241271058 for Complications of Stem Cell–Based Injections for Knee Osteoarthritis: A Systematic Review by Clara Riggle, Maddison McLellan, Hunter Bohlen and Dean Wang in HSS Journal®

sj-docx-3-hss-10.1177_15563316241271058 – Supplemental material for Complications of Stem Cell–Based Injections for Knee Osteoarthritis: A Systematic Review Supplemental material, sj-docx-3-hss-10.1177_15563316241271058 for Complications of Stem Cell–Based Injections for Knee Osteoarthritis: A Systematic Review by Clara Riggle, Maddison McLellan, Hunter Bohlen and Dean Wang in HSS Journal®

sj-docx-4-hss-10.1177_15563316241271058 – Supplemental material for Complications of Stem Cell–Based Injections for Knee Osteoarthritis: A Systematic Review Supplemental material, sj-docx-4-hss-10.1177_15563316241271058 for Complications of Stem Cell–Based Injections for Knee Osteoarthritis: A Systematic Review by Clara Riggle, Maddison McLellan, Hunter Bohlen and Dean Wang in HSS Journal®

sj-docx-5-hss-10.1177_15563316241271058 – Supplemental material for Complications of Stem Cell–Based Injections for Knee Osteoarthritis: A Systematic Review Supplemental material, sj-docx-5-hss-10.1177_15563316241271058 for Complications of Stem Cell–Based Injections for Knee Osteoarthritis: A Systematic Review by Clara Riggle, Maddison McLellan, Hunter Bohlen and Dean Wang in HSS Journal®

sj-docx-6-hss-10.1177_15563316241271058 – Supplemental material for Complications of Stem Cell–Based Injections for Knee Osteoarthritis: A Systematic Review Supplemental material, sj-docx-6-hss-10.1177_15563316241271058 for Complications of Stem Cell–Based Injections for Knee Osteoarthritis: A Systematic Review by Clara Riggle, Maddison McLellan, Hunter Bohlen and Dean Wang in HSS Journal®

sj-docx-7-hss-10.1177_15563316241271058 – Supplemental material for Complications of Stem Cell–Based Injections for Knee Osteoarthritis: A Systematic Review Supplemental material, sj-docx-7-hss-10.1177_15563316241271058 for Complications of Stem Cell–Based Injections for Knee Osteoarthritis: A Systematic Review by Clara Riggle, Maddison McLellan, Hunter Bohlen and Dean Wang in HSS Journal®
