# Supplementary material for: Complications of Stem Cell–Based Injections for Knee Osteoarthritis: A Systematic Review
Source: HSS J. 2024 Aug 16;21(4):476–84. doi: 10.1177/15563316241271058 (PMC11572451; doi:10.1177/15563316241271058)
Supplement: sj-docx-5-hss-10.1177_15563316241271058 – Supplemental material for Complications of Stem Cell–Based Injections for Knee Osteoarthritis: A Systematic Review [file sj-docx-5-hss-10.1177_15563316241271058.docx]

**Supplemental Table 1. Characteristics of Included Studies**

| **Author, Year** | **Parallel Intervention** | **Stem Cell Source** | **Volume Injected** | **Stem Cell Count** |
| --- | --- | --- | --- | --- |
| Mehling, 2020 | HA and/or PRP | SVF | N/A | N/A |
| Lamo-Espinosa, 2016 | HA and/or PRP | BMSC | 3 mL | 100 × 10^6^ |
| Lamo-Espinosa, 2020 | HA and/or PRP | BMSC | 3 mL | 100 × 10^6^ |
| Pak, 2013 | HA and/or PRP | SVF | 10 mL | N/A |
| Simunec, 2020 | HA and/or PRP | SVF | 7-37 mL | 3.5-17 × 10^6^ |
| Bastos, 2018 | HA and/or PRP | BMSC | 10 mL | 40 × 10^6^ |
| Hudetz, 2019 | None | SVF | 4-15 mL | N/A |
| LaPuente, 2020 | None | SVF | 7 mL | 15 × 10^6^ |
| Mautner, 2019 | None | BMAC and SVF | 8 mL and 5 mL | N/A |
| Themistocleous, 2018 | None | BMAC | 10 mL | N/A |
| Tsubosaka, 2020 | None | SVF | N/A | 2.5 × 10^7^ |
| Yokota, 2017 | None | SVF and ADMSC | 10 mL | N/A |
| Yokota, 2019 | None | SVF | 2.5 mL | 3 × 10^7^ |
| Goncars, 2019 | None | BMSC | N/A | N/A |
| Al-Najar, 2017 | None | BMSC | 5 mL | 30.5 × 10^6^ |
| Soler, 2016 | None | BMSC | 10 mL | 40 × 10^6^ |
| Orozoco, 2013 | None | BMSC | N/A | 40 × 10^6^ |
| Pers, 2016 | None | ADMSC | 5 mL | 2-50 x10^6^ |
| Chahal, 2019 | None | BMSC | 6.5 mL | 1-50 × 10^6^ |
| Rajput, 2018 | None | BMSC | N/A | N/A |
| Santoprete, 2021 | None | SVF | N/A | N/A |
| Michalek, 2019 | None | SVF | 1 mL-5mL | 15 × 10^6^ |
| Barfod, 2019 | None | SVF | N/A | N/A |
| Higuchi, 2020 | None | ADMSC | N/A | 1 × 10^4^ |
| Garza, 2020 | None | SVF | 3-4 mL | 1.5-3 × 10^7^ |
| Anz, 2020 | None | BMAC | 7 mL | N/A |
| Garay-Mendoza, 2018 | None | BMAC | 10 mL | 36.6 × 10^3^ |
| Freitag, 2019 | None | ADMSC | 3 mL | 100 × 10^6^ |
| Lu, 2019 | None | ADMSC | N/A | 5 × 10^7^ |
| Matas, 2019 | None | UCMSC | 3 mL | 20 × 10^6^ |
| Hernigou, 2018 | None | BMAC | 40 mL | 6.5 × 10^3^ |
| Lee, 2019 | None | ADMSC | 7 mL | 1 × 10^8^ |
| Shapiro, 2017 | None | BMAC | 15 mL | N/A |
| Hudetz, 2017 | None | SVF | 4-15 mL | N/A |
| Song, 2018 | None | ADMSC | 3 mL | 1-5 × 10^7^ |
| Khalifeh, 2019 | None | UCMSC | 10 mL | N/A |
| Schiavone, 2019 | Surgery | ADMSC | 10 mL | 2-50 × 10^6^ |
| Freitag, 2020 | Surgery | ADMSC | 3 mL | 50 × 10^6^ |
| Magnanelli, 2020 | Surgery | ADMSC | N/A | N/A |
| Cattaneo, 2018 | Surgery | ADMSC | N/A | N/A |
| Kim, 2018 | Surgery | ADMSC | 17 mL | N/A |
| Hong, 2019 | Surgery | SVF | 4 mL | N/A |
| Toan, 2020 | Surgery | BMSC | 10 mL | N/A |
| Jo, 2014 | Surgery | SVF | 5 mL | 1-5 × 10^7^ |
| Tran, 2019 | Surgery | SVF | 6 mL | 9-12 × 10^7^ |
| Wong, 2013 | Surgery | BMSC | 0.5-1 mL | N/A |
| Qiao, 2020 | Surgery | ADMSC | N/A | 5 × 10^7^ |
| Kim, 2016 | Surgery | BMAC | N/A | 4 × 10^6^ |
