# Supplementary material for: Complications of Stem Cell–Based Injections for Knee Osteoarthritis: A Systematic Review
Source: HSS J. 2024 Aug 16;21(4):476–84. doi: 10.1177/15563316241271058 (PMC11572451; doi:10.1177/15563316241271058)

Supplemental Table 3. Evaluation of randomized trial studies using Revised Cochrane Risk-of-Bias Tool


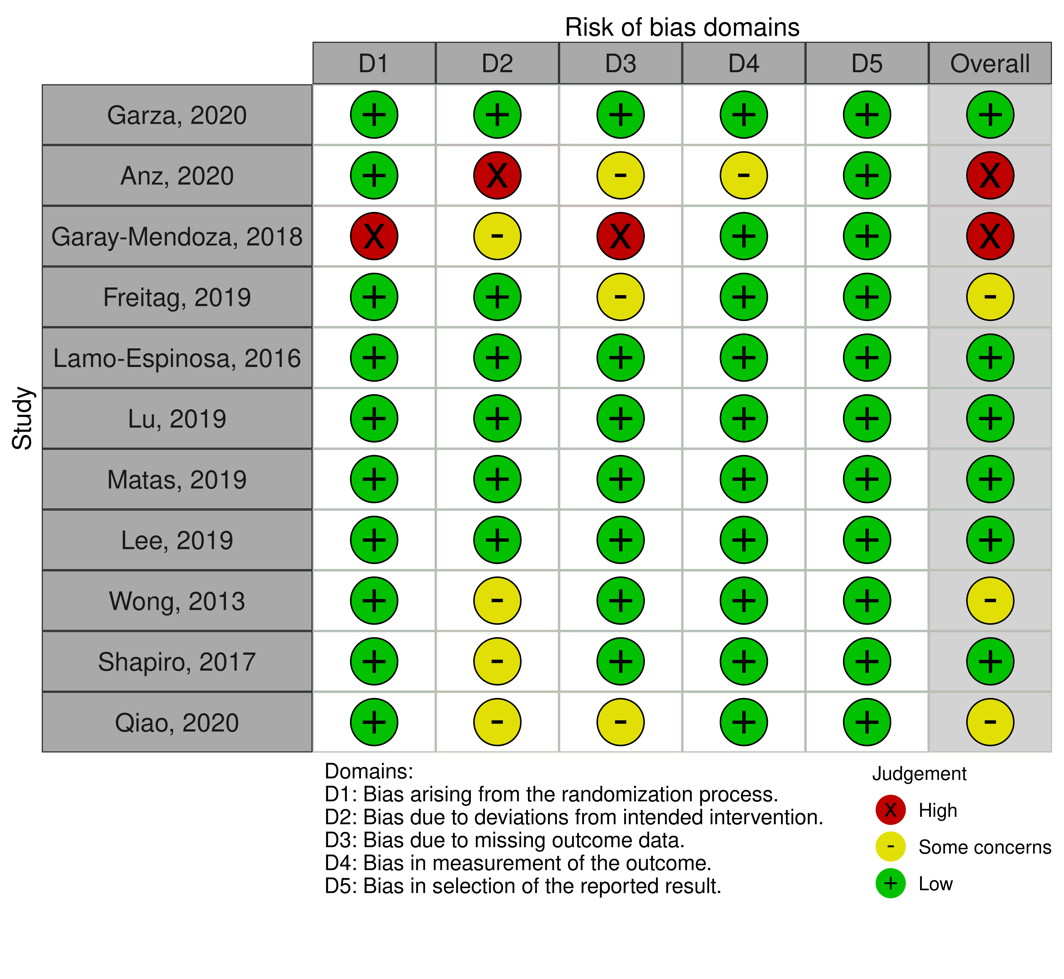

Supplement: sj-docx-7-hss-10.1177_15563316241271058 – Supplemental material for Complications of Stem Cell–Based Injections for Knee Osteoarthritis: A Systematic Review [file sj-docx-7-hss-10.1177_15563316241271058.docx]
